# Green Plant Pigment, Chlorophyllin, Ameliorates Non-alcoholic Fatty Liver Diseases (NAFLDs) Through Modulating Gut Microbiome in Mice

**DOI:** 10.3389/fphys.2021.739174

**Published:** 2021-10-26

**Authors:** You Yang, Xile Jiang, Stephen J. Pandol, Yuan-Ping Han, Xiaofeng Zheng

**Affiliations:** ^1^Center for Diabetes and Metabolism Research, Division of Endocrinology and Metabolism, West China Hospital, Sichuan University, Chengdu, China; ^2^The College of Life Sciences, Sichuan University, Chengdu, China; ^3^Department of Nutrition, West China College of Medicine, Sichuan University, Chengdu, China; ^4^Cedars-Sinai Medical Center, Los Angeles, CA, United States

**Keywords:** nonalcoholic fatty liver disease, sodium copper chlorophyllin, gut microbiota dysbiosis, endotoxin, intestinal barrier

## Abstract

Non-alcoholic fatty liver diseases (NAFLDs) along with metabolic syndrome and Type-2 diabetes (T2D) are increasingly prevalent worldwide. Without an effective resolution, simple hepatic steatosis may lead to non-alcoholic steatohepatitis (NASH), characterized by hepatocyte damage, chronic inflammation, necrosis, fatty degeneration, and cirrhosis. The gut microbiome is vital for metabolic homeostasis. Conversely, dysbiosis contributes to metabolic diseases including NAFLD. Specifically, diet composition is critical for the enterotype of gut microbiota. We reasoned that green pigment rich in vegetables may modulate the gut microbiome for metabolic homeostasis. In this study, C57BL/6 mice under a high fat diet (HFD) were treated with sodium copper chlorophyllin (CHL), a water-soluble derivative of chlorophyll, in drinking water. After 28 weeks of HFD feeding, liver steatosis was established accompanied by gut microbiota dysbiosis, intestinal impairment, endotoxemia, systemic inflammation, and insulin resistance. Administration of CHL effectively alleviated systemic and intestinal inflammation and maintained tight junction in the intestinal barrier. CHL rebalanced gut microbiota in the mice under high fat feeding and attenuated hepatic steatosis, insulin resistance, dyslipidemia, and reduced body weight. Fecal flora transplants from the CHL-treated mice ameliorated steatosis as well. Thus, dietary green pigment or the administration of CHL may maintain gut eubiosis and intestinal integrity to attenuate systemic inflammation and relieve NASH.

## Introduction

Non-alcoholic fatty liver diseases (NAFLDs) are defined as fatty liver disorders while the subjects are without significant alcohol consumption. NAFLDs are often prevalent together with Type-2 diabetes (T2D), obesity, and metabolic syndrome. The global prevalence of NAFLD is estimated to be 20–25% in the general population, with the highest in the Middle East (32%), South America (30%), followed by Asia (27%), North America (24%) Europe (23%) and Africa (13%) (Chalasani et al., 2018). About 20% of NAFLD in simple steatosis may further progress into non-alcoholic steatohepatitis (NASH), featured as persistent liver inflammation, hepatic fat degeneration, liver injury and necrosis, and hepatic fibrosis and cirrhosis, while some may eventually progress into hepatocellular carcinoma (HCC) (Pais et al., 2013; Calzadilla Bertot and Adams, 2016). In fact, NASH-associated cirrhosis has become the second major challenge for adult liver transplantation in the United States (Wong et al., 2015). The biological pathogenesis of NAFLD is poorly understood. It is believed that NAFLD comes from complex interactions among excessive calorie intake, malnutrition, genetic susceptibility, environmental factors, and insulin resistance (Arab et al., 2017). The interaction of these risk factors may lead to abnormal lipid metabolism and excessive lipid accumulation in hepatocytes, which can further convert to NAFLD. Changing lifestyles, such as the control of body weight and diet balance, may delay or prevent the development of NASH from simple steatosis (Johnson and George, 2010; Vilar-Gomez et al., 2015). Persistent NASH with chronic liver injury, inflammation, and cirrhosis should be subjected to clinical treatment. However, at present, although there are numerous potential drugs in clinical trials for NASH treatment, none of them is approved by FDA or international medical authorities. Despite insulin resistance and T2D are closely related to NDALD, none of the drugs for T2D was approved for NASH treatment.

The gut microbiome is essential for maintaining physiological homeostasis in many ways (Wong et al., 2015; Leung et al., 2016). In germ-free mice with high-fat feeding, the extent of hepatic steatosis was significantly lower than the control, indicating the contribution of the gut microbiome for NAFLD (Rabot et al., 2010). Transplantation of the gut microbes from the mice with hyperglycemia and insulinemia to the germ-free recipient mice could lead to hepatic steatosis in association with reduced Bacteroides in the gut (Roy et al., 2013). Some bacterial species in the human gut were known for association with NAFLD (Kolodziejczyk et al., 2018). The intestinal flora of patients with NASH is often abundant with proteus, enterobacter, escherichia (Zhu et al., 2013), and few Bacteroides (Boursier et al., 2016).

Correlation analysis of NAFLD-associated parameters and the abundance of bacterial species in mice fed with low-fat and high-fat diet showed an association between *Lactobacillus gasseri* and *Lactobacillus taiwanensis* with lipidic droplets in the liver (Zeng et al., 2013). Short-term use of non-absorbable antibiotic rifaximin improved the liver function of patients with NASH (Gangarapu et al., 2015). Similarly, long-term antibiotic treatment leads to the decrease of intestinal bacterial growth, which is also related to the improvement of liver function (Madrid et al., 2001).

Intestinal innate immunity is composed of intercellular tight junctions, mucus produced by goblet cells, antimicrobial defenses from Paneth cells, and innate and adaptive immune cells (Turner, 2009). Delicate interactions and balance among intestinal microbiota, intestinal epithelial cells, and intestinal mucosal system are very important for maintaining intestinal permeability and tissue homeostasis (Peterson and Artis, 2014). Patients with NAFLD often have intestinal dysfunction or increased intestinal permeability (Miele et al., 2009; Luther et al., 2015). A clinical study showed that circulating bacterial endotoxin is directly related to the biogenesis of NAFLD (Yang et al., 1997; Sharifnia et al., 2015), which is further confirmed by animal work showing that high levels of lipopolysaccharides (LPS) can induce obesity and insulin resistance (Cani et al., 2007). On the contrary, *Tlr4* knockout in mice protected the mice from liver injury, inflammation, and lipid accumulation (Rivera et al., 2007).

Chlorophyll is the most abundant plant pigment on the earth, functioning for photosynthesis in plants and algae. Sodium copper chlorophyllin (CHL) is one of the most widely used semi-synthetic and water-soluble forms of chlorophylls that is widely used as a food and beverage color agent (Tumolo and Lanfer-Marquez, 2012). CHL is also used as a therapeutic agent. Sodium copper CHL was used to control odor and promoting wound healing (Telgenhoff et al., 2007; Stephens et al., 2015). A number of *in vivo* and *in vitro* studies have confirmed that CHL had antigenotoxicity, antioxidant, and anticancer activities (Wogan et al., 2012). The partial hydrophobic nature of chlorophyll and its binding capacity with carcinogens are believed to have health benefits (Dashwood and Guo, 1993; Sarkar et al., 1996). The antigenotoxicity and anticancer effects of CHL are thought to be mediated by scavenging free radicals or porphyrin rings that form complexes with planar carcinogens (Fahey et al., 2005; Linnewiel et al., 2009).

In this study, we examined the potential role of CHL in the alleviation of diet-induced hepatic steatosis and metabolic disorders. We found that CHL can modulate gut microbiota in the mice subjected to high fat feeding, and attenuate hepatic steatosis, insulin resistance, dyslipidemia, and reduced body weight. Thus, dietary green pigment and pharmacological administration of CHL may help to maintain gut eubiosis and intestinal integrity to attenuate systemic inflammation, and such agents may be used for relieving non-alcoholic steatohepatitis as well.

## Materials and Methods

### High-Fat-Diet Feeding Induced Non-alcoholic Fatty Liver Diseases (NAFLD) and Treatment Regimens

All animal experimental procedures in this study have complied with guidelines as outlined in the “Guide for the Care and Use of Laboratory Animals” (National Research Council, USA). The animal protocols were approved by the Institutional Animal Care and Use Committee, the College of Life Sciences, Sichuan University. Briefly, 5-week-old C57BL/6 male mice of SPF grade (Beijing HFK Bioscience) were maintained in a controlled environment (12:12 light-dark cycle) with free access to food and water. After 1 week of adaptation, the mice were randomly divided into three groups: (1) control group, by which mice were fed with ANI93 chow for 28 weeks, (2) high fat diet (HFD) group, of which 65% calorie in the chow was derived from animal fat, (3) high fat diet feeding plus chlorophyllin treatment (HFD+CHL), by which the mice under HFD feeding were treated with CHL in drinking water (30 mg/L) from the 17th week for consecutive 12 weeks (*n* = 8–10 for each group). Calorie intake, body weight, and fasting blood glucose were monitored accordingly. The liver, blood samples, the ileum tissues, and fecal pellets were collected for analysis.

### Fecal Flora Transplantation (FFT)

After high fat feeding for 20 weeks, the recipient mice were divided into 3 groups and subjected to fecal transplant. Group #1, mice under HFD feeding were subjected to the transplant of fecal microbes derived from control AIN93 chow, labeled as F-C. Group #2, mice under HFD feeding were transplanted with fecal microbes, derived from high fat feeding, as F-HFD. Group #3, mice under HDF feeding were subjected to the transplant of fecal microbes derived from the CHL-treated mice, labeled as F-CHL. For fecal flora transplant, fresh donor feces as mentioned above from CHL-treated mice were collected. After washing to remove the debris and CHL in the feces, about 4 × 10^8^ colony-forming unit (CFU) bacteria in 0.1 ml suspension were administered by gavage to the recipient mice. FFT was repeated 3 times per week for consecutive 6 weeks (*n* = 6 for each group), in a total of 26 weeks.

### Histological Analysis

Liver tissues were fixed in 4% of paraformaldehyde, and hematoxylin and eosin (H&E) staining were used for histologic analysis. Liver fibrosis was determined by Masson's Trichrome staining and the sizes of fibrotic septa were quantitated *via* densitometry analysis. The intestinal mucus layer was measured with a periodic acid Schiff (PAS) red stain kit (ab150680, Abcam, MA, USA), following the instruction of the manufacturer. Lymphocyte infiltration in the liver was determined by immunohistochemical staining for anti-CD3 (Cat. 16669; Abcam, MA, USA). Colorimetric images for H&E and Masson's Trichrome staining were captured *via* Nikon eclipse Ti-U microscope (Nikon, Japan). Tight junctions in the ileum were examined by immunofluorescent staining with anti-occludin (Cat. SC5562; Santa Cruz Biotechnology, TX, USA) and all images were captured *via* the Leica TCS SP5 II system (Leica, Germany).

### Reverse Transcription-Quantitative Real-Time PCR Analysis

The liver and ileum tissues were homogenized in 1 ml Trizol and total RNA was extracted. The RNA was converted to cDNA *via* the Transcriptor First Strand cDNA Synthesis Kit (Cat. 04897030001; Roche, MA, USA). The quantitative PCR (qPCR) system contained 2 μl cDNA, 0.2 μl forward primer, and 0.2 μl reverse primer (200 nM), 5 μl MIX, and diethylpyrocarbonate (DEPC) water was added to 10 μl. The reaction was performed with a Bio-Rad machine Cfx96. The primer sequence information is listed in Table 1. The relative microRNA (mRNA) expression was normalized in the expression of RPL-19.

**Table 1:** The list of reverse transcription quantitative real-time PCR (RT-qPCR) primers for mice.

**Gene**	**Forward primer (5^′^−3^′^)**	**Reverse primer (5^′^−3^′^)**
TNF-α	TGGGACAGTGACCTGGACTGT	TTCGGAAAGCCCATTTGAGT
IL-1β	TCGCTCAGGGTCACAAGAAA	CATCAGAGGCAAGGAGGAAAAC
MUC2	GCTCGGAACTCCAGAAAGAAG	GCCAGGGAATCGGTAGACAT
GADPH	GCACAGTCAAGGCCGAGAAT	GCCTTCTCCATGGTGGTGAA
Arginase1	GAACCCAACTCTTGGGAAGAC	GGAGAAGGCGTTTGCTTAGTT
Occludin	ATGTCCGGCCGATGCTCTC	TTTGGCTGCTCTTGGGTCTGTAT
ZO-1	ACCCGAAACTGATGCTGTGGATAG	AAATGGCCGGGCAGAACTTGTGTA
Claudin-2	CCTTCGGGACTTCTACTCGC	TCACACATACCCAGTCAGGC
IL-6	CTTCCATCCAGTTGCCTTCTTG	AATTAAGCCTCCGACTTGTGAAG
CoIIα1	GCTCCTCTTAGGGGCCACT	CCACGTCTCACCATTGGGG
α-SMA	GACGCTGAAGTATCCGATAGAACACG	CACCATCTCCAGAGTCCAGCACAAT
TIMP1	GCATGGACATTTATTCTCCACTGT	TCTCTAGGAGCCCGATCTG
TGF-β	CTTCAGCTCCACAGAGAAGA	GACAGAAGTTGGCATGGTAG

### Gut Microbiota Analysis

SYBR green-based qPCR analysis of 16S rRNA genes was used to quantitate the relative abundance of gut bacteria. Fecal microbe DNA was extracted using a stool DNA kit (Omega, China). The qPCR system contained 2 μl DNA, 0.2 μl forward primer, 0.2 μl reverse primer (200 nM), 5 μl MIX, and DEPC water was added to 10 μl. Then, it was analyzed with the Bio-Rad Cfx96 and the value was expressed as the percentage of common bacterial readouts as the internal reference. The accuracy of the qPCR-based 16 rDNA analysis was previously validated by sequencing the PCR products. The primers for bacterial qPCR analysis are listed in Table 2.

**Table 2:** The list of 16S recombinant DNA (rDNA) primers for microbiota.

**Gene**	**Forward primer (5^′^−3^′^)**	**Reverse primer (5^′^−3^′^)**
All bacteria	ACTCCTACGGGAGGCAGCAG	ATTACCGCGGCTGCTGG
Firmicutes	GGAGTATGTGGTTTAATTCGAAGCA	AGCTGACGACAACCATGCAC
Bacteriods	GGARCATGTGGTTTAATTCGATGAT	AGCTGACGACAACCATGCAGG
ε-proteobacteria	TGGTGTAGGGGTAAAATCCG	AGGTAAGGTTCTTCGYGTATC
Desulfovibrio	CCGTAGATATCTGGAGGAACATCAG	ACATCTAGCATCCATCGTTTACAGC
Prevotella	GAAGGTCCCCCACATTG	CAATCGGAGTTCTTCGTG
Enterobacteriacease	CATTGACGTTACCCGCAGAAGAAGC	CTCTACGAGACTCAATCTTGC

### Metabolic Parameters

*The* protocols for intra-peritoneal glucose tolerance test (IPGTT) and homeostatic model assessment of insulin resistance (HOMA-IR), fasting glucose, intestinal permeability, and histological analysis were previously reported (Zhu et al., 2017). Mice were fasted for 6 or 4 h for GTTs or ITTs, respectively. Glucose (1 g/kg body weight, 20% glucose solution) or insulin (1 U/kg body weight) was injected intraperitoneally. Blood glucose was measured by a glucose meter (Accu-Chek Active, Roche, Germany) with 5 μl of blood was collected from the tip of the tail vein. Plasma insulin concentration was measured by ELISA kit (DRE30417; RB China). HOMA-IR is determined as glucose concentration x insulin concentration divided by 22.5. Limulus amebocyte extract kit (Chinese Horseshoe Crab Reagent Manufactory, Xiamen, China) was used to detect plasma lipopolysaccharide (LPS) content. Plasma TNF-alpha levels were measured by ELISA kit (Mercodia, Uppsala, Sweden; DRE30030, China). Plasma levels of low-density lipoprotein cholesterol (LDLC), high-density lipoprotein cholesterol (HDLC), total cholesterol (CHOL), total bile acids, and alanine transaminase (ALT) levels were measured by the automation instrument in the Chengdu Public Health and Clinical Medical Center.

### Statistical Analysis

Data recording, processing, and calculating were completed using Microsoft Excel 2003 and GraphPad Prism 5.0. Data were plotted as a mean ± SEM. Comparisons across groups were assessed using a Student's-*t*-test or one-way ANOVA, followed by Tukey multiple comparison testing. Statistical significance was performed as ^*^P < 0.05 or ^**^P < 0.01.

## Results

### Chlorophyllin Ameliorates Body Weight Gain of the Mice Under High Fat Feeding

**Figure 1:**
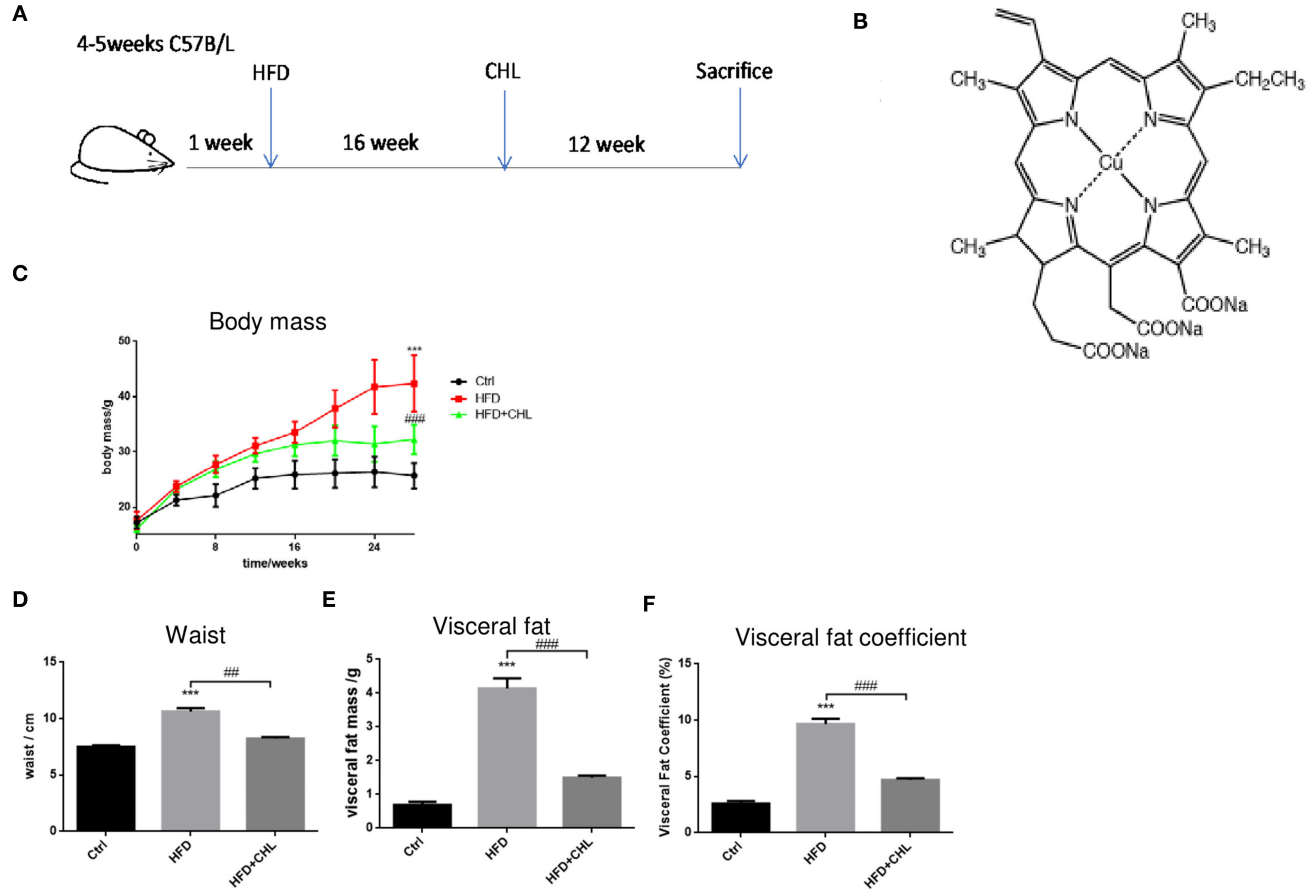
Chlorophyllin (CHL) attenuates body weight gain by high fat feeding in mice. **(A)** B57BL/6 male mice were randomly divided into three groups: control group, fed with AIN93 chow; high fat diet (HFD) group, by which 65% calorie comes from animal fat; HFD+ CHL group, by which the mice were fed with HFD for 16 weeks followed by adding CHL (30 mg/L) in drinking water for another 12 weeks. **(B)** Structure of CHL. **(C)** Changes in body mass. **(D)** Changes in waist size. **(E)** Total visceral fat. **(F)** Visceral fat co-efficiency, visceral fat/body weight. *n* = 6–8 for each group. Values are mean ± SEM; ***p < 0.001 vs. Ctrl group; ^##^*p* < 0.01, ^###^*p* < 0.001 vs. HFD group.

Mice in C57BL/6 genetic background were fed with control AIN93 chow (Control) or high fat diet (HFD) for 28 weeks. Another group of HFD mice was given chlorophyllin (CHL) in drinking water (30 mg/L) starting from the 17th week for additional 12 weeks (HFD+CHL) (Figures 1A,B). As a common feature of high fat feeding, the mice gained their body weight starting from week 4 and doubled their weight to about 40 g in 28 weeks (Figure 1C). Administration of the green pigment in drinking water significantly reduced the body weight gain by the high fat feeding group (HFD+CHL). We also measured the waist, visceral fat, and visceral fat coefficient, which were all attenuated by CHL administration as shown in Figures 1D–F. Mice normally drink 3–5 ml water, which is about 0.09–0.15 mg CHL daily. Spinach contains chlorophyll at 0.85 mg/g, which is equivalent to 0.12–0.15 g of spinach per day for the mice. For a human with 60 kg body weight, the dose of CHL would be at 0.18–0.3 g daily.

### Chlorophyllin Improves Metabolic Parameters of the Mice Under High Fat Feeding

**Figure 2:**
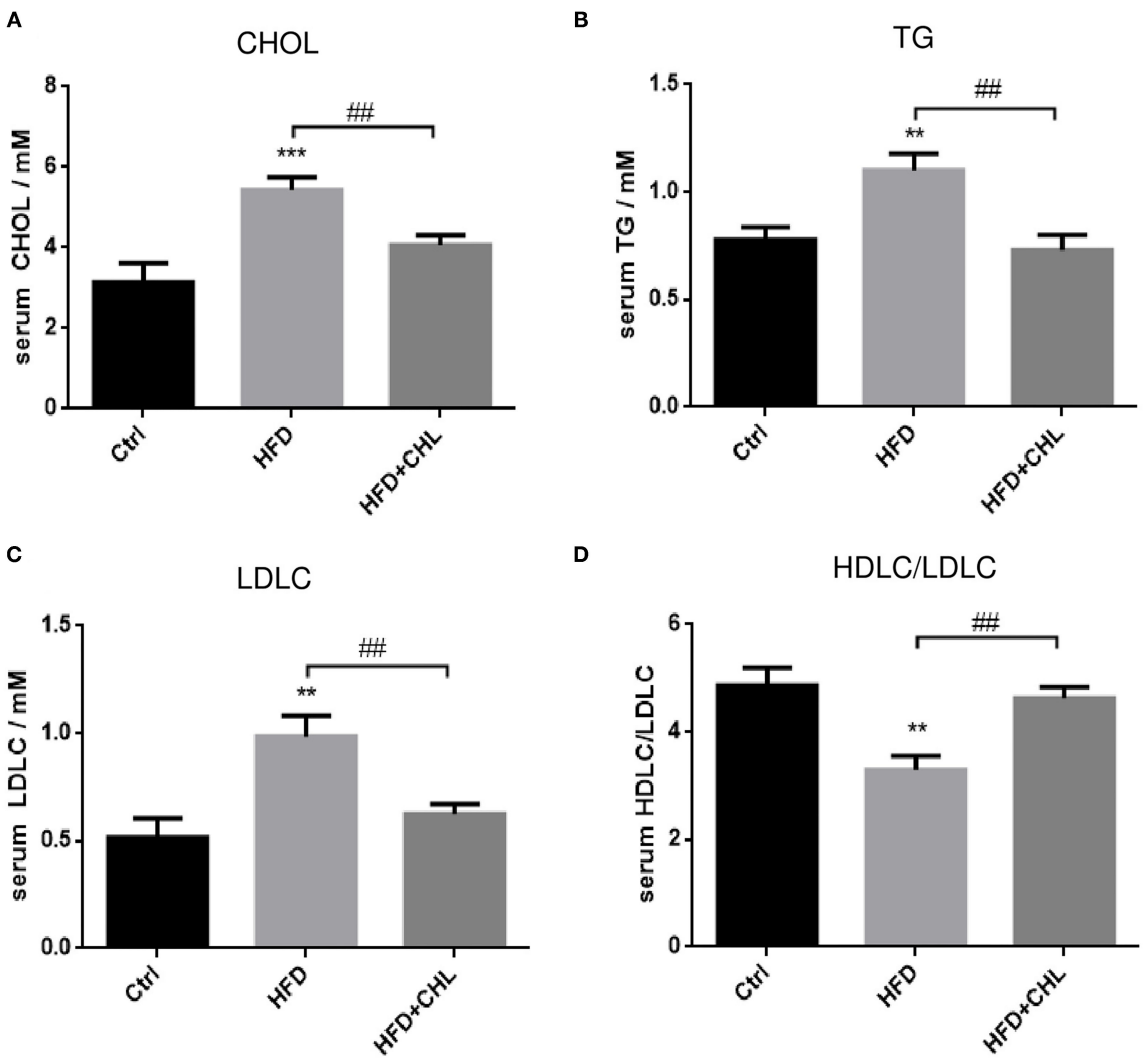
CHL improves the metabolic parameters of the mice fed with an HFD. The experimental condition is described in Figure 1. In the end, the metabolic parameters of mice were measured. **(A)** Plasma levels of cholesterol. **(B)** Plasma triglycerides. **(C)** Plasma low-density lipoprotein cholesterol (LDLC) levels. **(D)** The ratio of high-density lipoprotein cholesterol (HDLC)/LDLC and *n* = 6–8 for each group. Values are mean ± SEM; **p < 0.01, ***p < 0.001 vs. Ctrl group; ^##^*p* < 0.01 vs. HFD group.

Then we extended the assessment by including other metabolic parameters. After 28 weeks of high fat feeding, plasma levels of cholesterol, triglycerides, and LDL-C in the mice were all remarkably increased (Figure 2). Conversely, the green pigment in the drinking water statistically reduced these metabolic parameters in agreement with alleviated metabolic abnormalities, such as body weight and visceral fat. Importantly, the ratio of (HDL-C)/(LDL-C) is restored by CHL treatment.

### Chlorophyllin Improves Hepatic Steatosis and Liver Functions

**Figure 3:**
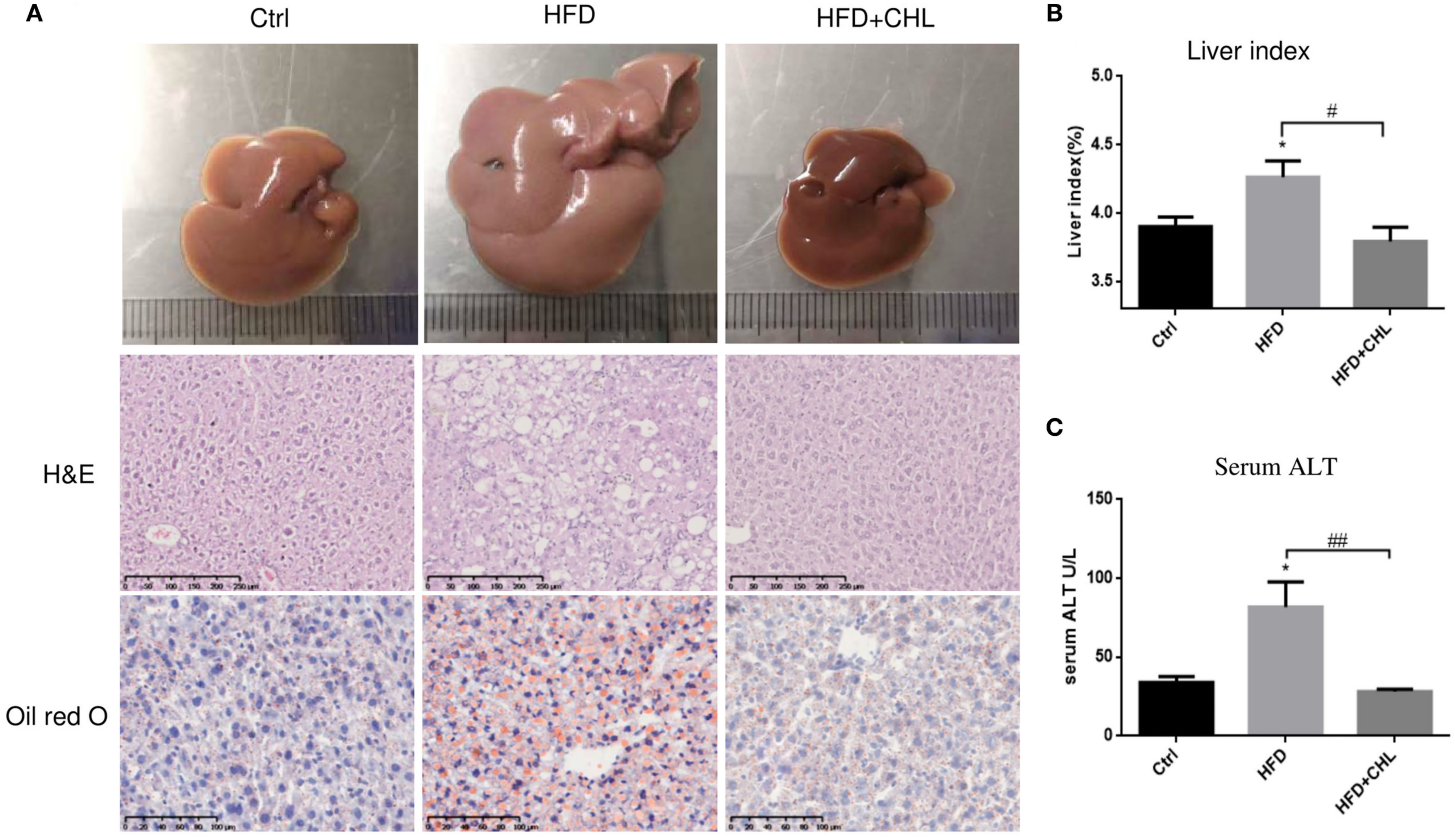
CHL ameliorates hepatic steatosis and liver functions of the mice fed with an HFD. **(A)** The experimental condition is described in Figure 1. In the end, images of the liver, histochemical staining by hematoxylin and eosin (HandE), and Oil Red O were determined. **(B)** Liver index, liver mass vs. body mass. **(C)** Serum alanine transaminase (ALT) levels and *n* = 6–8 for each group. Values are mean ± SEM; *p < 0.05 vs. Ctrl group; ^#^*p* < 0.05, ^##^*p* < 0.01 vs. HFD group.

It is well-known that overweight and central obesity are tightly connected with the key features of NAFLD (Fock and Khoo, 2013; Dietrich and Hellerbrand, 2014; Milić et al., 2014). Indeed, high fat feeding for 28 weeks led to enlarged liver mass, a key feature of NAFLD in the early phase (Figure 3A). Parenchymal damage in hepatocytes as determined by the histological assessment, a crucial character of fat degeneration in hepatocytes, was also evident by HFD feeding. Hepatic steatosis was validated by Oil Red O staining. As shown, CHL in drinking water reduced the liver mass/liver index, and improved steatosis (Figure 3B). Liver injury as indicated by elevation of serum alanine transaminase (ALT) was significantly ameliorated by CHL in drinking water in agreement with improved histological findings (Figure 3C).

### Chlorophyllin Improves Hepatic Inflammation Exerted by High Fat Feeding

**Figure 4:**
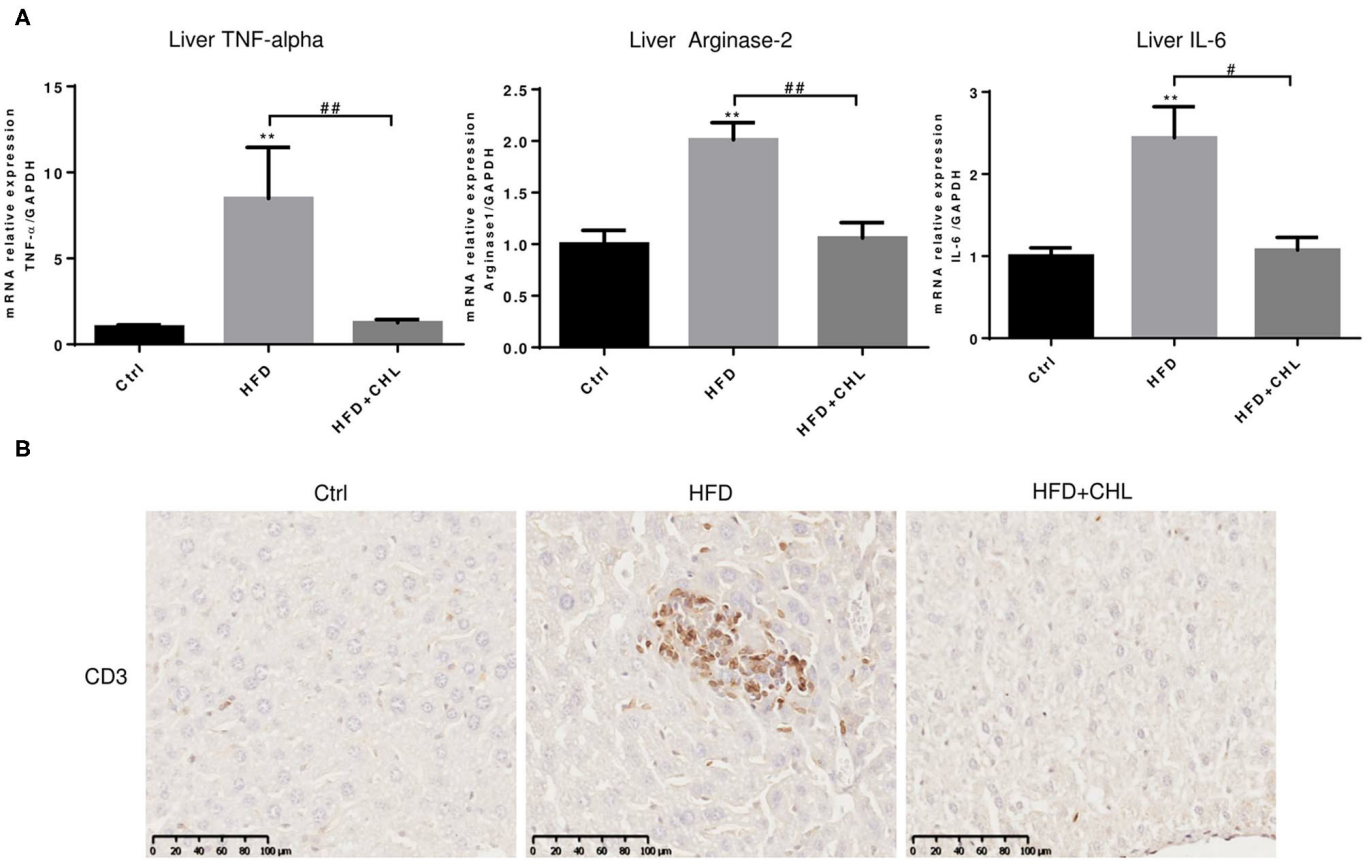
CHL suppresses hepatic inflammation of the mice fed with an HFD. **(A)** The experimental condition is described in Figure 1. In the end, the liver inflammation was measured by expression of tumor necrosis factor-α (TNF-α, Arginase-2, and Interleukin-6 (IL-6) by reverse transcription-quantitative real-time PCR RT-qPCR) analysis. **(B)** Infiltration of lymphocytes was monitored by immunohistochemical staining for CD3+ cells in the liver and *n* = 6–8 for each group. Values are mean ± SEM; **p < 0.01 vs. Ctrl group; ^#^*p* < 0.05, ^##^*p* < 0.01 vs, HFD group.

Persistent inflammation is a hallmark for the initial prognosis of simple steatosis into nonalcoholic steatohepatitis (NASH). Interleukin 6 (IL-6) is highly relevant to hepatic malignancy, an ominous sign of NASH (Tsuchida et al., 2018; Anstee et al., 2019; Malehmir et al., 2019), while arginase-2 is related to persistent inflammation and skewed immune responsiveness (Delyea et al., 2018). We measured the hepatic expression of inflammatory cytokines. As shown, hepatic tumor necrosis factor-α **(**TNF-α, IL-6, and arginase-1 at their microRNA (mRNA) levels were increased by high fat feeding (Figure 4A). As shown in our data, CHL thoroughly suppressed the hepatic expression of these inflammatory mediators, and the data is in agreement with the reduced liver injury and improved liver function (Figure 3). Indeed, infiltration of lymphocytes, as marked by CD3+ staining, was obvious in the parenchyma of liver tissue by the mice under HFD feeding, which was thoroughly suppressed by the administration of CHL (Figure 4B), in line with the reduced expression of inflammatory cytokines.

### Chlorophyllin Ameliorates Hepatic Fibrosis Caused by High Fat Feeding

**Figure 5:**
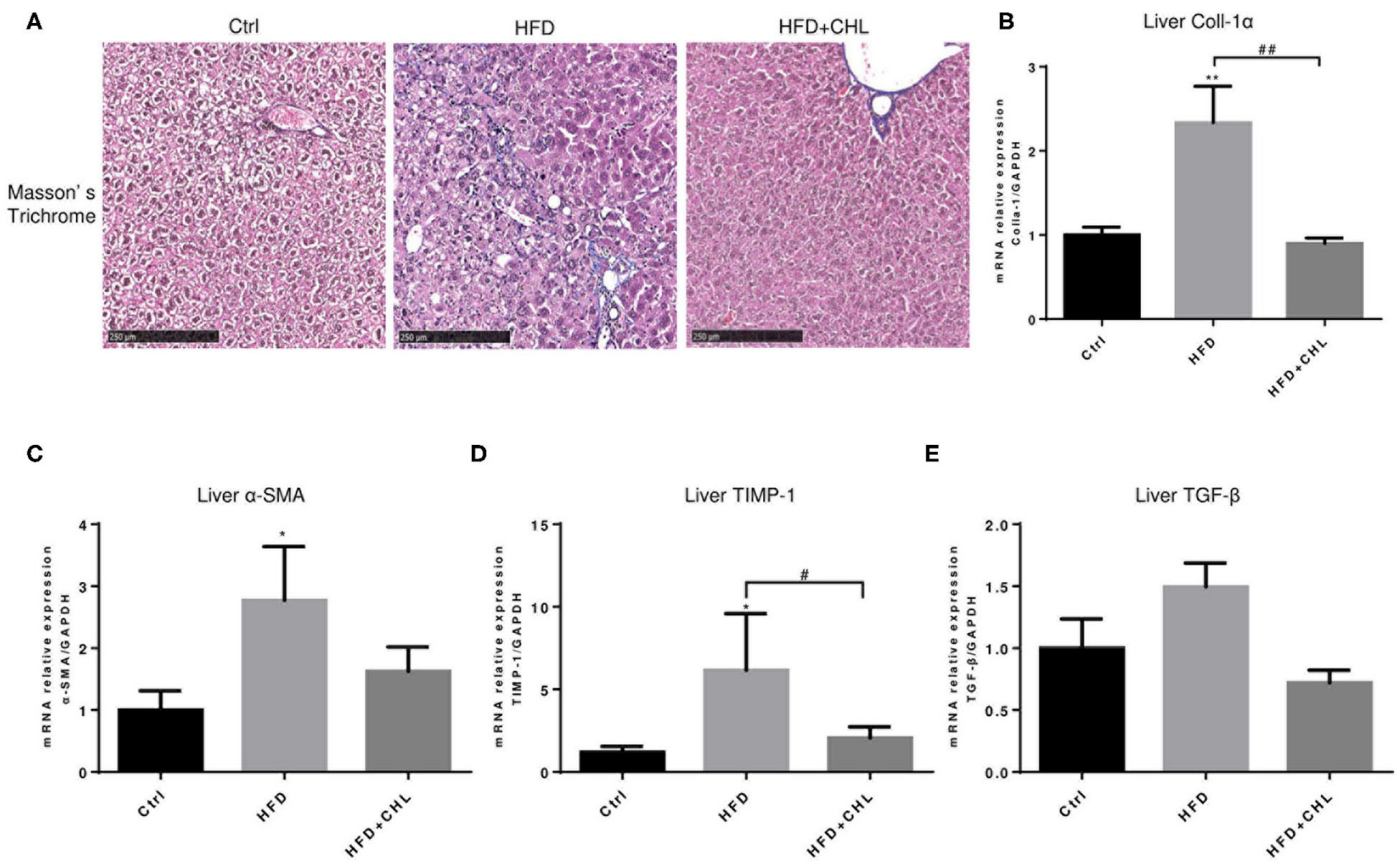
CHL improves liver fibrosis of the mice fed with an HFD. **(A)** The experimental condition is described in Figure 1. The liver fibrosis was determined by Masson's Trichrome staining. Hepatic expression of **(B)** Type-I collagen alpha, **(C)** alpha-smooth actin, **(D)** tissue inhibitor of matrix metalloproteinase-1, and **(E)** transforming growth factor beta-1 was determined by RT-qPCR analysis and *n* = 6–8 for each group. Values are mean ± SEM; *p < 0.05, **p < 0.01 vs. Ctrl group; ^#^*p* < 0.05, ^##^*p* < 0.01 vs. HFD group.

Liver fibrosis, as a consequence of hepatic injury and inflammation, is essential for wound healing and as a key marker of NASH (Enomoto et al., 2015). Masson's Trichrome staining showed that liver fibrosis is moderately appeared by high fat feeding for 28 weeks, and such fibrosis was ameliorated by the administration of CHL (Figure 5A). Expression of Type-1 alpha collagen in the liver, as measured by RT-qPCR analysis, was significantly increased by high fat feeding, but suppressed by the administration of CHL (Figures 5B–E). In a similar pattern, CHL treatment also suppressed the expression of TGF-beta1, and alpha-smooth actin, the key genes for liver fibrosis. Moreover, TIMP1, a tissue inhibitor for matrix metalloproteinases (MMPs) is also suppressed by CHL treatment, indicating that CHL may promote MMP-mediated resolution of liver fibrosis.

### Chlorophyllin Improves Insulin Responsiveness

**Figure 6:**
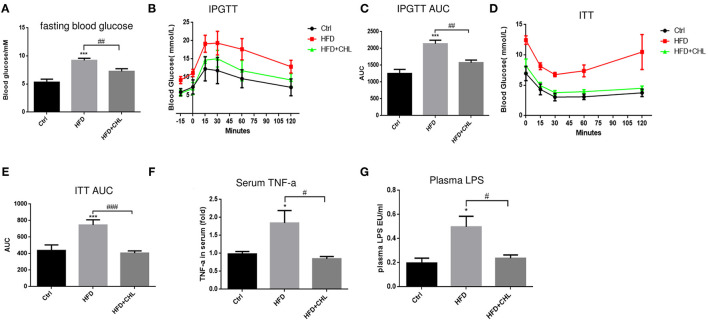
CHL improves insulin responsiveness and liver functions of the mice fed with an HFD. **(A)** Fasting blood glucose was measured by the mice as mentioned in the section, Materials and methods. **(B)** Intraperitoneal glucose tolerance test (IPGTT) of the three groups of mice. **(C)** Aura under the curve (AUC) of IPGTT was determined. **(D)** The insulin tolerance test (ITT) was measured for the three groups of mice. **(E)** AUC of ITT was determined. **(F)** Serum ALT levels were determined by ELISA analysis. **(G)** Plasma lipopolysaccharide (LPS) was determined by Limulus amebocyte lysate (LAL) analysis and *n* = 6–8 for each group. Values are mean ± SEM; *p < 0.05, ***p < 0.001 vs. Ctrl group; ^#^*p* < 0.05, ^##^*p* < 0.01, ^###^*p* < 0.001 vs. HFD group.

Type-2 diabetes (T2D) is a major driving force for hepatic steatosis. In fact, the accumulation of hepatic triglycerides in hepatocytes serves as a compensation mechanism to reduce persistent hyperglycemia, due to systemic insulin resistance. As shown in Figure 6A, fasting glucose was evidently increased in the mice of high fat feeding, which was suppressed by CHL treatment. Intraperitoneal glucose tolerance test (IPGTT) showed glucose intolerance, an indicator of insulin resistance in the mice of high fat feeding (Figures 6B,C). Likewise, the insulin tolerance test (ITT) indicated insulin resistance was built up in the mice under high fat feeding. Conversely, CHL treatment mostly resolved the resistance (Figures 6D,E). Systemic inflammation is known as a leading cause of insulin resistance (Jiao et al., 2018; Jia et al., 2020). In fact, increased plasma TNF-α levels as an indicator for systemic inflammation in the mice under HFD feeding were mostly resolved by CHL treatment (Figure 6F). Endotoxin, a prominent intestinal promoter for systemic inflammation and insulin resistance for hepatic steatosis, is elevated in the mice under HFD feeding, was suppressed by CHL treatment (Figure 6G).

### Chlorophyllin Modulates the Gut Microbiome

**Figure 7:**
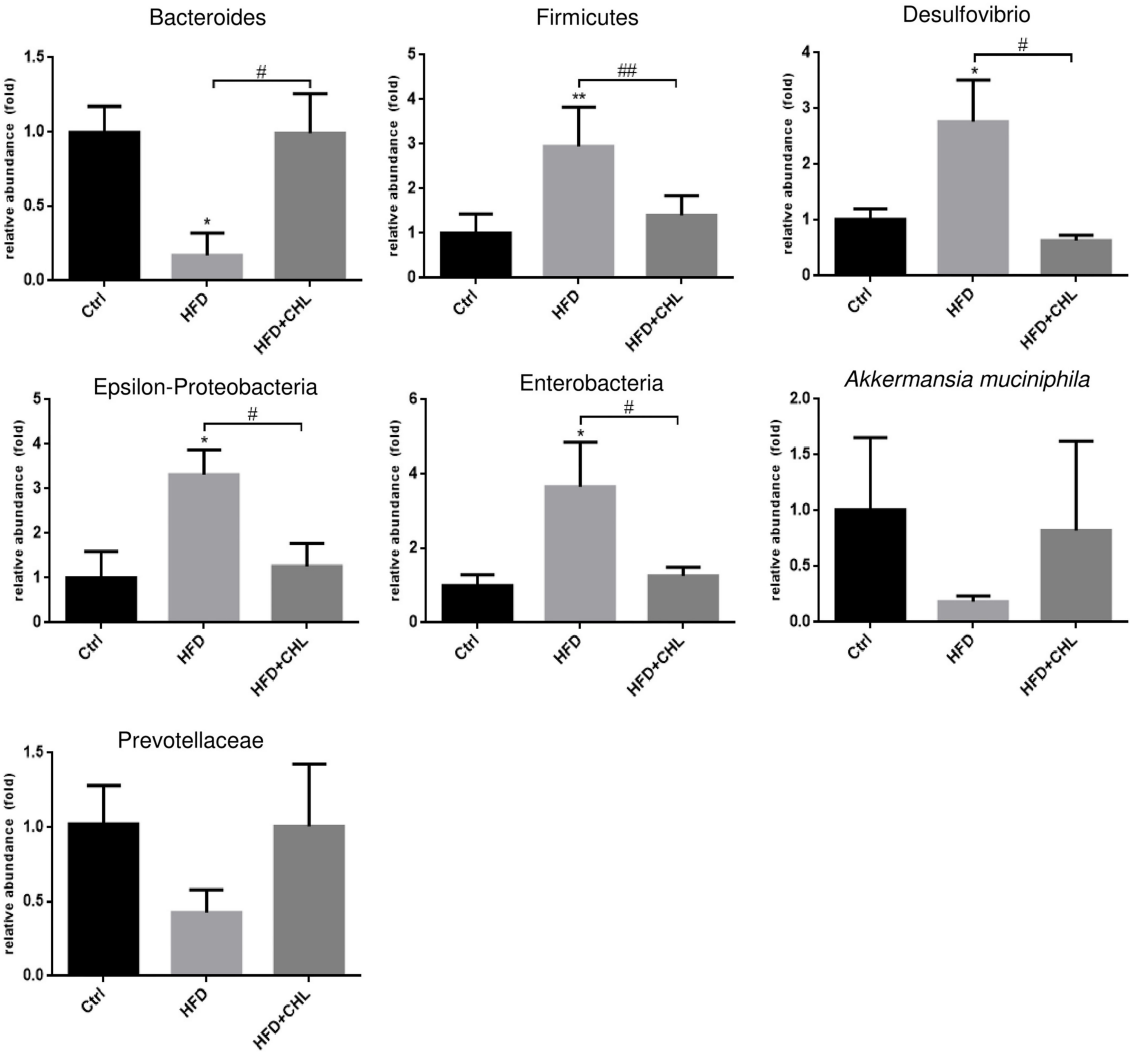
Chlorophyllin (CHL) balances the gut microbiome of the mice fed with an HFD. Fresh fecal pellets were collected for extraction of bacteria DNA. Specific microbes, including Bacteroides, Firmicutes, Desulfovibrio, Epsilon-Proteobacteria, Enterobacteria, *Akkermansia muciniphila*, were determined by qPCR as mentioned in the section, Materials and methods and *n* = 6–8 for each group. Values are mean ± SEM; *p < 0.05, **p < 0.01 vs. Ctrl group; ^#^*p* < 0.05, ^##^*p* < 0.01 vs. HFD group.

Increased plasma endotoxin levels in the mice of HFD feeding, and the suppression by CHL treatment indicate that the green pigment may modulate the gut microbiome, leading to the downregulation of systemic inflammation and restoration of insulin sensitivity, which ultimately may resolve the hepatic steatosis. As shown in Figure 7, intestinal Bacteroides, the major Gram-negative microbes, were downregulated by long-term HFD feeding, which was in agreement with increased plasma endotoxin. CHL treatment thoroughly restored intestinal Bacteroides in line with the downregulation of plasma endotoxin. Firmicutes, another major gut microbe that is responsible for fermentation and production of short chain fatty acid for obesity, were increased in their abundance by HFD feeding. Conversely, CHL treatment suppressed the abundance of Firmicutes, which is in line with the reduction of body mass. Desulfovibrio, a sulfate-reducing bacterium, was increasingly presented in the gut by HFD feeding, and CHL treatment also suppressed the microbe. Similarly, epsilon-proteobacteria and enterobacteria, which were increased by the mice under HFD feeding, were suppressed by CHL treatment. *Akkermansia muciniphila*, a major species of intestinal symbiotic and being regarded as a marker of eubiosis, and Prevotellaceae were both downregulated by HFD feeding as previously reported in our study (Su et al., 2016). CHL treatment unequivocally restored these microbes. All these results demonstrate that the green pigment can modulate the gut microbiome, and the restored microbiota may consequently suppress NAFLD.

### Chlorophyllin Attenuates Intestinal Inflammation and Preserves Innate Immunity

**Figure 8:**
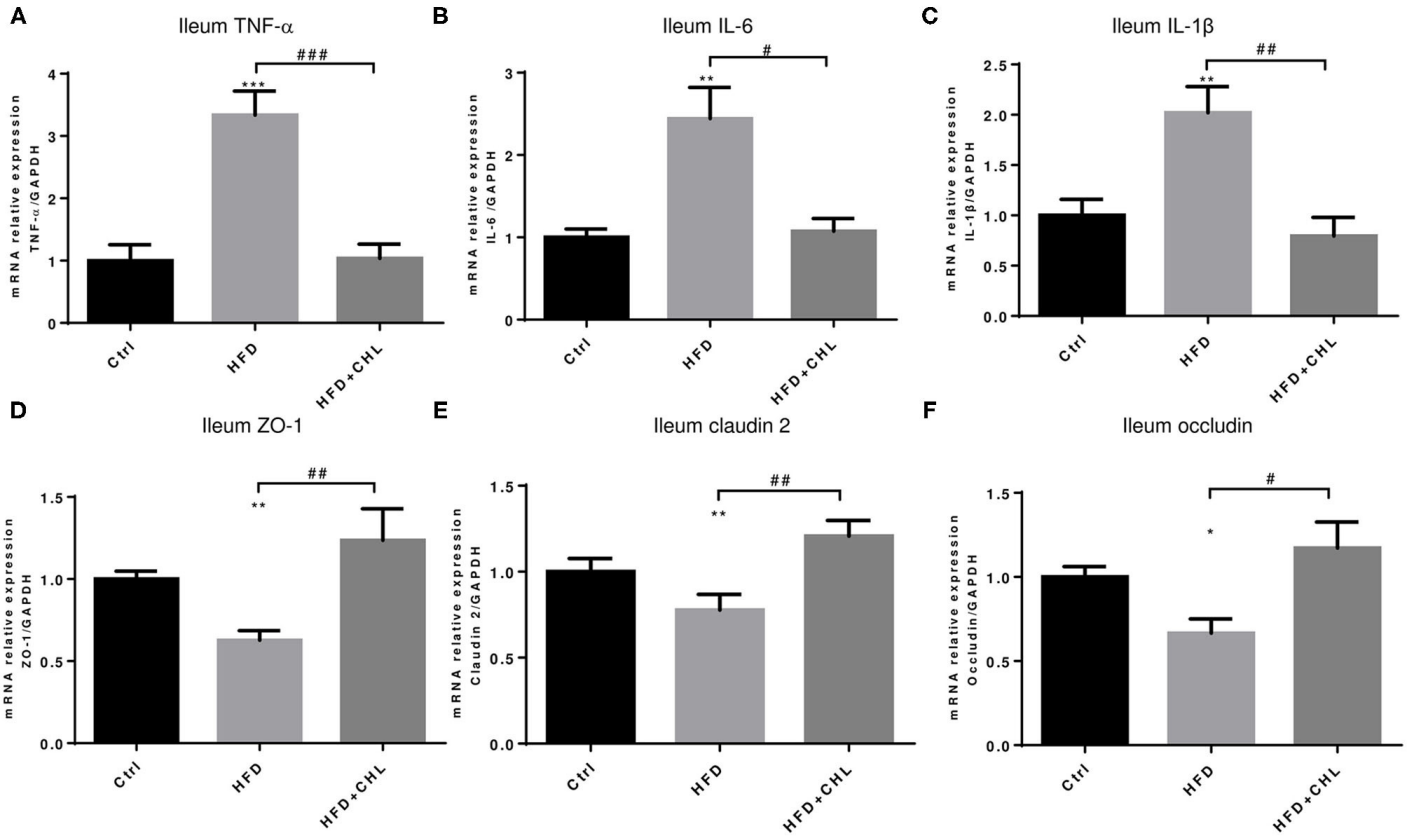
CHL improves small intestinal innate immunity and suppresses intestinal inflammation of the mice fed with an HFD. **(A)** The experimental condition is described in Figure 1. The microRNA (mRNA) levels of TNF-α in the ileum were determined by RT-qPCR analysis. **(B)** The mRNA of interleukin 6 (IL-6) levels of the ileum. **(C)** The mRNA levels of IL-1b in the ileum. **(D)** ZO1 expression in the ileum. **(E)** The mRNA levels of Claudin 2 expression in the ileum. **(F)** The mRNA levels of occludin expression in the ileum and *n* = 6–8 for each group. Values are mean ± SEM; *p < 0.05, **p < 0.01, ***p < 0.001 vs. Ctrl group; ^#^*p* < 0.05, ^##^*p* < 0.01, ^###^*p* < 0.001 vs. HFD group.

**Figure 9:**
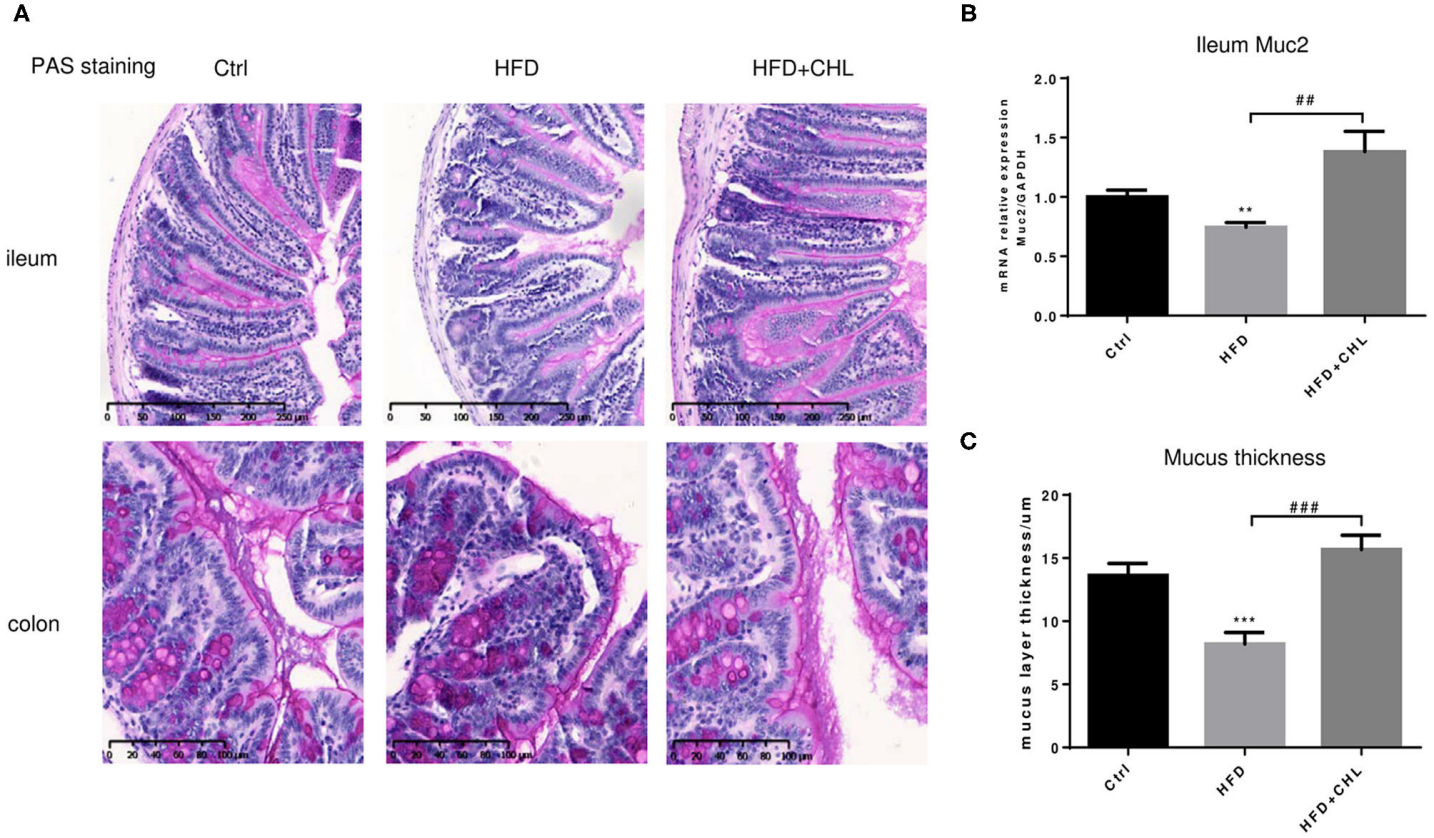
CHL improves the small intestinal mucus of the mice fed with an HFD. **(A)** The experimental condition is described in Figure 1. Periodic acid–Schiff (PAS) staining of the ileum and colon. **(B)** Muc2 expression in the ileum was determined by RT-qPCR analysis. **(C)** The thickness of mucus layers of the ileum, as an average of three views for each mouse. Values are mean ± SEM; **p < 0.01, ***p < 0.001 vs. Ctrl group; ^##^*p* < 0.01, ^###^*p* < 0.001 vs. HFD group.

Balancing the gut microbiome by CHL treatment also indicates an inverse impact on intestinal innate immunity. Damaged intestinal epithelia in the mice under HFD feeding may cause intestinal leakage for increased endotoxemia. Here, we showed that the HFD-induced intestinal inflammation, as indicated by the expression of TNF-α, IL-6, and IL-1β in the ileum of the mice, was suppressed by CHL treatment (Figures 8A–C). Conversely, downregulated expression of tight junction proteins in the ileum, including ZO-1, claudin 2, and occludin by HFD feeding, was restored by CHL treatment (Figures 8D–F). Moreover, the mucus of the ileum and colon regions was examined by Periodic acid–Schiff (PAS) staining. As shown in Figures 9A,C, HFD feeding led to the damage of the mucus layer and shortening of villi, while CHL treatment reversed the gut injury. Muc2, a major mucin gene for the intestine, was suppressed by HFD feeding, in line with the damaged intestinal epithelia. CHL treatment restored Muc2 expression, presumably through reduced intestinal inflammation (Figure 9B).

### Fecal Transplant of Gut Microbe Derived From the CHL-Treated Mice Can Ameliorate Metabolic Disorders and Hepatic Steatosis

**Figure 10:**
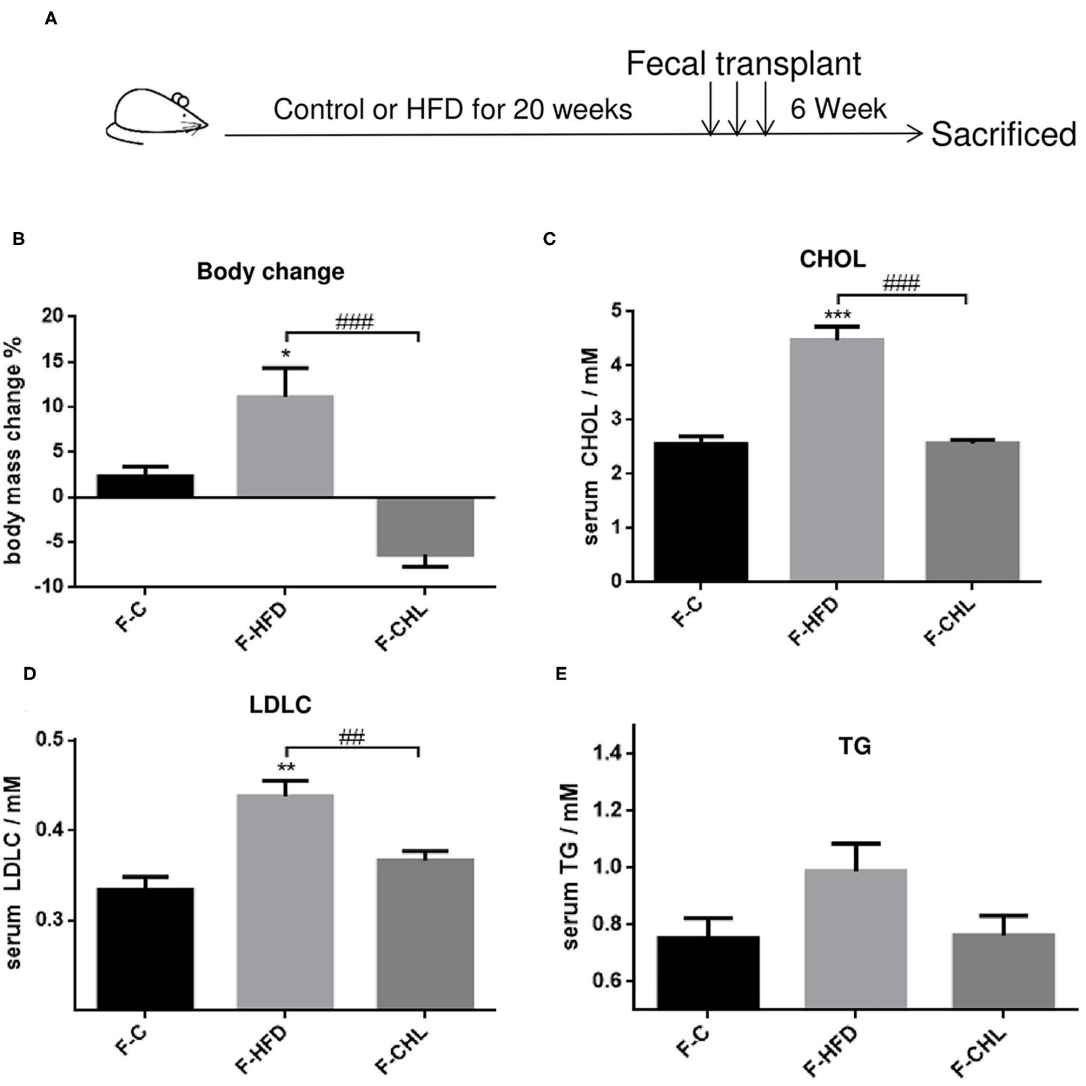
Fecal flora transplant from CHL-treated mice improves metabolic parameters of the mice fed with an HFD. **(A)** Experiment design for fecal flora transplant. **(B)** Bodyweight of the recipient mice at the end of the experiment. **(C)** Plasma cholesterol levels of the recipient mice at the end of the experiment. **(D)** Plasma levels of low-density lipoprotein cholesterol (LDLC) by the recipient mice at the end of the experiment. **(E)** Plasma levels of triglycerides and *n* = 6 for each group. Values are mean ± SEM; *p < 0.05, **p < 0.01, ***p < 0.001 vs. Ctrl group; ^##^*p* < 0.01, ^###^*p* < 0.001 vs. HFD group.

**Figure 11:**
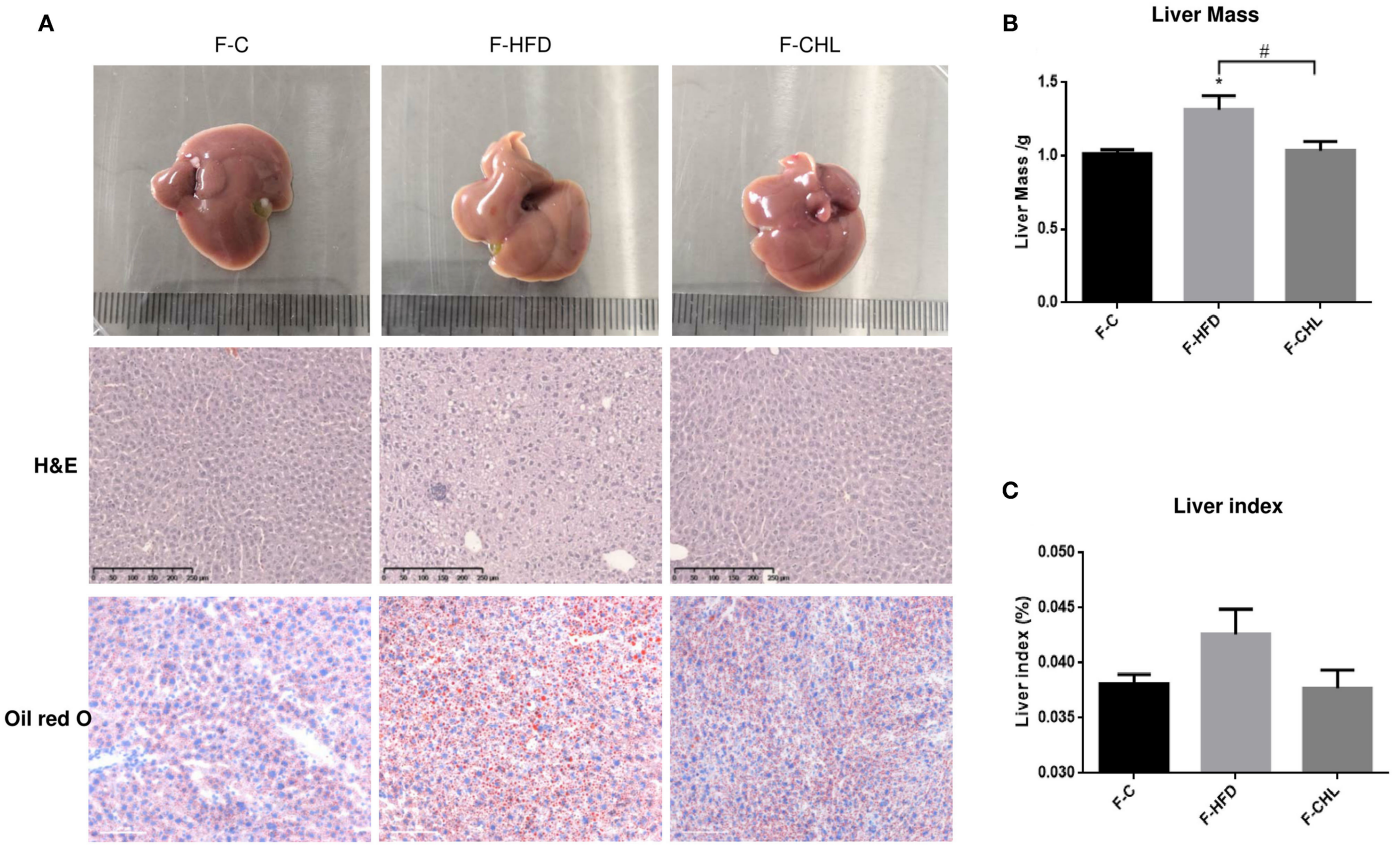
Fecal flora transplant from CHL-treated mice attenuates hepatic steatosis of the mice fed with an HFD. **(A)** Fecal transplant, as mentioned in the section, Materials and methods. Liver images, HandE staining of the liver, and Oil Red O staining of the liver tissues. **(B)** Liver mass. **(C)** Liver index and *n* = 6 for each group. Values are mean ± SEM; *p < 0.05 vs. Ctrl group; ^#^*p* < 0.05 vs/ HFD group.

**Figure 12:**
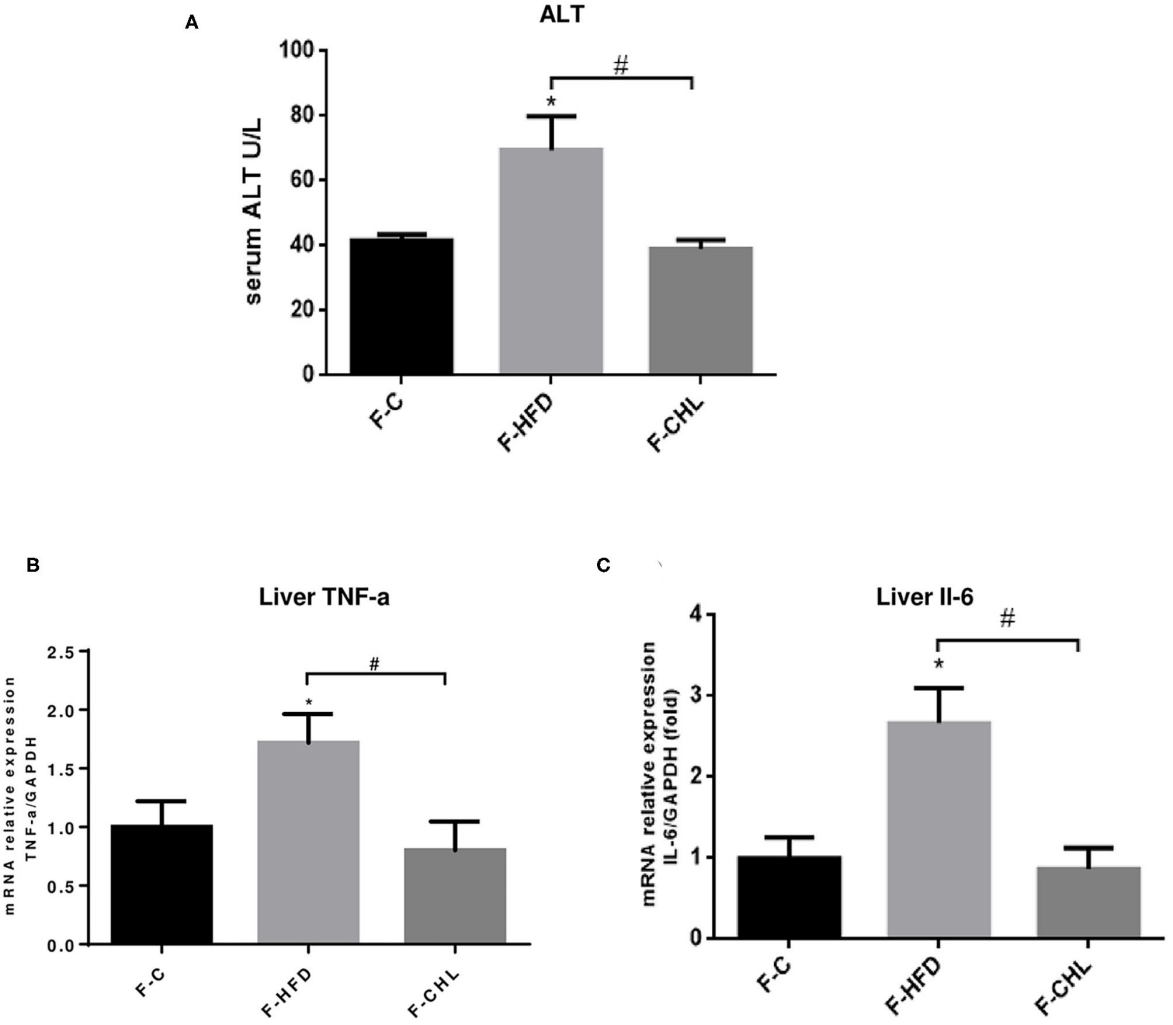
Fecal flora transplant from CHL-treated mice improves liver functions and suppresses liver inflammation of the mice fed with an HFD. **(A)** Fecal transplant, as mentioned in the section, Materials and methods. Plasma ATL levels of the recipient mice at the end of the experiment. **(B)** Hepatic expression of TNF-a was determined by RT-qPCR analysis. **(C)** Hepatic expression of Interleukin 6 (IL-6) was determined by RT-qPCR analysis and *n* = 6 for each group. Values are mean ± SEM; *p < 0.05 vs. Ctrl group; ^#^*p* < 0.05 vs. HFD group.

To validate the causal roles of the gut microbiome in the CHL-exerted improvement of fatty liver and metabolic disorders, we applied fecal transplant. After high fat feeding for 20 weeks, the recipient mice were divided into 3 groups and subjected to fecal transplant. For fecal flora transplants, fresh donor fecal pellets were collected from the HFD mice under CHL treatment for 6 weeks. After washing, bacteria at 4 x 10^8^ CFU were administered by gavage to the recipient mice, repeating 3 times per week for consecutive 6 weeks (Figure 10A). As shown in Figure 10B, the fecal flora derived from the CHL-treated mice could suppress the gain of body weight by the recipients under HFD feeding. Metabolic parameters including plasma cholesterol, LDL-C, and triglycerides were also improved by the fecal transplant (Figures 10C–E). Similarly, fatty liver, as measured by Oil Red O staining, and liver mass, were mostly resolved by the microbes derived from the CHL treated mice (Figure 11). Liver inflammation, as the expression of TNF-α and IL-6, was suppressed by the fecal transplant, leading to the restoration of liver functions as evidenced by the reduction of plasma ALT levels (Figure 12).

## Discussion

Non-alcoholic fatty liver diseases as a group of prominent metabolic diseases are often related to poor eating habits and malnutrition in addition to excessive calorie intake. This study uncovered that the green pigment of plants, in the format of chlorophyllin (CHL), can modulate the gut microbiome, which consequently suppresses intestinal and systemic inflammation and relieves hepatic steatosis. In particular, oral administration of CHL can upregulate the intestinal abundance of Bacteroides and downregulates the population of Firmicutes, a key feature of gut eubiosis for physiological homeostasis against metabolic syndrome and NAFLD (Turnbaugh, 2006; Turnbaugh et al., 2009; Chatelier et al., 2013). Gut dysbiosis is a major driving force for various metabolic disorders including obesity, T2D, and NAFLD (Leung et al., 2016; Fukui, 2019; Tilg et al., 2019; Aron-Wisnewsky et al., 2020). While the mechanism of CHL in modulating the gut microbiome is unknown, it may either directly impact gut microbes or indirectly regulate intestinal epithelial cells as we previously reported, showing suppression of cellular IKK activation (Zheng et al., 2018). Moreover, CHL may sequestrate the dietary toxin or the gut-microbe-produced chemicals and promote their excretion into feces. Above all, restoration of Bacteroidetes, the major Gram-negative bacteria, is associated with the downregulation of plasma endotoxin, suggesting that CHL may suppress the death of the gut microbes, which consequently reduces the influx of lipopolysaccharide (LPS) into the portal vein, leading to alleviation of systemic inflammation. In clinical studies and animal works, increased plasma LPS is tightly related to the incidence of NAFLD (Yang et al., 1997; Sharifnia et al., 2015). It is well-known that LPS from the gut microbes is a prominent proinflammation driving force, causing insulin resistance and glucose intolerance, which may further promote the excessive synthesis of triglycerides for hepatic steatosis (Soares et al., 2010).

The disconnection or malfunction of the gut-liver axis plays an important role in the pathogenesis of NAFLD in many ways, such as impairment of intestinal barrier, direct bacterial translocation, and an influx of endotoxin (Kolodziejczyk et al., 2018). Under physiological conditions, tight junctions of intestinal epithelial cells prevent bacteria from entering the intestinal mucosa and blood (Turner, 2009). Intestinal mucus produced by goblet cells and alpha-defensins made by Paneth cells protects the host from gut microbes. Previous work from this group found that vitamin D receptor is highly expressed in the distal region of the small intestine and vitamin D signaling can protect the host from steatosis (Su et al., 2016). In particular, our previous work showed that vitamin D signal, through upregulation of Paneth cell defensins and tight junction genes of the host, can suppress the influx of endotoxin from portal vein to the liver, which consequently reduces systemic and local inflammation, and attenuates insulin resistance and relieves steatosis. Moreover, another work in this lab showed that sequestration of intestinal endotoxin *via* cholestyramine, cationic polymeric resins, can profoundly resolve systemic inflammation, restore insulin sensitivity, and attenuate hepatic steatosis and liver fibrosis (Zhu et al., 2017). The green pigment of plants, including CHL, on the other hand, may serve as probiotics to modulate the gut microbiome for host metabolic homeostasis. Importantly, this study implies that consumption of green vegetables may help to prevent NAFLD and supplemental of CHL may relieve NAFLD, which should be further examined in clinical trials.

## Data Availability Statement

The original contributions presented in the study are included in the article/supplementary files, further inquiries can be directed to the corresponding author/s.

## Ethics Statement

The animal study was reviewed and approved by Institutional Animal Care and Use Institutional Animal Care and Use Committee, the College of Life Sciences, Sichuan University.

## Author Contributions

YY, Y-PH, and XZ conceived the project and designed the experiments. YY and XJ performed the experiments and analyzed the data. YY and Y-PH prepared the figures and drafted the manuscript. XJ, SP, and XZ revised the manuscript. All authors contributed to the article and approved the submitted version.

## Funding

The work was supported by the Natural Science Foundation of China (NSFC), #31571165 and #31771288 to Y-PH, #82070846 to XZ, and the National Institutes of Health (USA) P01CA163200, P50 AA011999, and P01DK098108 to SP.

## Conflict of Interest

The authors declare that the research was conducted in the absence of any commercial or financial relationships that could be construed as a potential conflict of interest.

## Publisher's Note

All claims expressed in this article are solely those of the authors and do not necessarily represent those of their affiliated organizations, or those of the publisher, the editors and the reviewers. Any product that may be evaluated in this article, or claim that may be made by its manufacturer, is not guaranteed or endorsed by the publisher.

## Abbreviations

CHL: Chlorophyllin
NAFLDs: non-alcoholic fatty liver diseases
NASH: non-alcoholic steatohepatitis
TNF-alpha: tumor necrosis factor-alpha.
